# The v8-10 variant isoform of CD44 is selectively expressed in the normal human colonic stem cell niche and frequently is overexpressed in colon carcinomas during tumor development

**DOI:** 10.1080/15384047.2023.2195363

**Published:** 2023-04-02

**Authors:** Bruce M. Boman, Vignesh Viswanathan, Caroline O. B. Facey, Jeremy Z. Fields, James W. Stave

**Affiliations:** a Center for Translational Cancer Research, Helen F. Graham Cancer Center & Research Institute, Newark, DE, USA; b Department of Biologic Sciences, University of Delaware, Newark, DE, USA; c Jefferson Kimmel Cancer Center, Thomas Jefferson University, Philadelphia, PA, USA; d Department of Radiation Oncology, Stanford University School of Medicine, Stanford, CA, USA; e Department of Cancer Research and Innovation, CA*TX Inc, Princeton, NJ, USA; f Department of Cancer Research and Innovation, Strategic Diagnostics Inc, Newark, DE, USA

**Keywords:** CD44 protein, CD44v8-10, CD44 variant isoforms, cancer stem cells, colorectal cancer

## Abstract

CD44 protein and its variant isoforms are expressed in cancer stem cells (CSCs), and various CD44 isoforms can have different functional roles in cells. Our goal was to investigate how different CD44 isoforms contribute to the emergence of stem cell (SC) overpopulation that drives colorectal cancer (CRC) development. Specific CD44 variant isoforms are selectively expressed in normal colonic SCs and become overexpressed in CRCs during tumor development. We created a unique panel of anti-CD44 rabbit genomic antibodies to 16 specific epitopes that span the entire length of the CD44 molecule. Our panel was used to comprehensively investigate the expression of different CD44 isoforms in matched pairs (*n* = 10) of malignant colonic tissue and adjacent normal mucosa, using two (IHC & IF) immunostaining approaches. We found that: *i*) CD44v8–10 is selectively expressed in the normal human colonic SC niche; *ii*) CD44v8–10 is co-expressed with the SC markers ALDH1 and LGR5 in normal and malignant colon tissues; *iii*) colon carcinoma tissues frequently (80%) stain for CD44v8–10 while staining for CD44v6 was less frequent (40%). Given that CD44v8–10 expression is restricted to cells in the normal human colonic SC niche and CD44v8–10 expression progressively increases during CRC development, CD44v8–10 expression likely contributes to the SC overpopulation that drives the development and growth of colon cancers. Since the CD44 variant v8–10 epitope is located on CD44’s extracellular region, it offers great promise for targeted anti-CSC treatment approaches.

## Introduction

CD44 is widely used in tumor biology and clinical oncology as a stem cell (SC) marker. Indeed, a PubMed search shows that there are 7,314 published papers on CD44 and stem cells^1^. Also, our PubMed search shows that there are 1,104 published papers on CD44 and colorectal cancer (CRC). Thus, there is a substantial body of scientific literature reporting that CD44 is a cancer stem cell (CSC) marker, and functions as a key regulator of cancer stemness, self-renewal, tumor initiation, and metastasis. Moreover, CD44 is widely used, alone or in combination, with other SC markers, to isolate or enrich CSCs using fluorescence-activated-cell-sorting (FACS) of cells from patient tissues, xenograft tumor tissues, or tumor cell cultures. Indeed, we^2^ and others^3^ reported that CSCs isolated using antibodies against the standard form of CD44 (CD44s) protein have tumor-initiating ability in immuno-deficient mice. The problem is that we also found that CD44s is not only expressed in normal and malignant colonic SCs but also, it is expressed in proliferative non-SCs^2^. This finding shows that CD44s is not a specific marker for colonic SCs since it also marks proliferative cells. Accordingly, our objective was to determine whether any of the various isoforms generated by the alternative splicing of CD44 are more specific for identifying colonic SCs.

It is important to understand how CD44 and its various isoforms play a role in the stemness of colonic SCs because we^2^ and others^3–12^ have shown that SC overpopulation drives colon cancer development and growth. Indeed, CD44 is an SC marker that has been widely studied for its role in the development of many cancer types. Since CD44 is a transmembrane protein that binds to hyaluronic acid (HA), which is expressed by stromal cells, overexpression of CD44 may promote local invasion through altered HA binding and metabolism^13^. And, through its ability to promote epithelial-mesenchymal transition (EMT)^14,15^, CD44 plays a role in the generation of CSCs^16,17^. We found that CD44 can track SC overpopulation during CRC development^2^. And, CD44+ CRC cells have tumor-initiating ability in immuno-deficient mice^2,3,18^. This result suggests that CD44 may be a prognostic marker for CRC patient outcomes. However, clinical studies on CD44 expression in CRCs revealed that CD44 is not a prognostic marker based on CRC patient survival^16^. Still, assessing CD44 expression is complicated because many variant CD44 isoforms can be expressed on cancer cells and some, but not all, have predictive value. For example, CD44v6 was shown to be a useful marker for tumor progression and prognosis in colon carcinoma patients^19^. But, in other cancers, an increased level of CD44 expression did not correlate with patient outcomes^20^. Interestingly, in rectal cancer, it is the lack of CD44v6 expression that correlated with early recurrence^21^. The situation is complicated even further because in several studies that use CD44 antibodies, the CD44 isoform that the antibody was designed to recognize was not specified^22^.

The biology of CD44 is not fully understood because several hundred isoforms can be produced in cells through alternative splicing. The multiple CD44 isoforms are generated by insertion of alternative exons at specific sites that encode CD44’s extracellular domain^23^. The transcripts that emerge from this complex alternative splicing leads to many functionally distinct isoforms. And, the biology of a number of these variants has not been determined. The variant isoforms of CD44 (CD44v) are comprised of exons 6–15 spliced at different sites within the non-variant isoform. Interestingly, the expression of specific CD44 isoforms appears to be involved in the progression of human tumors. Indeed, some predominant CD44 isoforms have been identified and shown to be biologically important in various cancers^19,24^. For example, larger size isoforms have been found to be expressed in CRC (e.g. CD44v6)^19,25^ and in pancreatic cancer (e.g. CD44v8–10)^26^. In CRCs, the CSCs were found to express CD44v6, which was essential for CSC migration and generation of metastatic tumors^15^. Moreover, the expression of CD44v6 was associated with advanced tumor grade and poor overall survival^19^. CD44v6 expression also predicted tumor response of CRC patients to treatment^27^. In prostate cancer cells, different CD44 isoforms are expressed depending on the metastatic site of origin^23^. In breast cancer cells, the switch from CD44v6 to CD44s is essential for EMT and tumor progression^28^. Thus, because CD44 proteins, especially CD44 variant isoforms, are expressed in CSCs, and various CD44v isoforms can have different functional roles^22,29^, there are many gaps in our knowledge regarding how CD44 isoforms contribute to the emergence of CSCs. We hypothesize that specific CD44 variant isoforms are selectively expressed in normal colonic SCs and become overexpressed during CRC development. Accordingly, our study generated a unique panel of specific CD44 antibodies that react to different epitopes along the entire length of the CD44 molecule in order to comprehensively investigate the expression of different CD44 isoforms in normal colonic epithelium and in colon carcinomas.

## Methods

*Procurement of Colon Tissue Samples*. All the tissue sections used in this study were obtained through the Tissue Procurement Core Facility at the Helen F. Graham Cancer Center & Research Institute. Our study was approved by the IRB at Christiana Care Health Systems. All tissue samples were de-identified and stripped of all direct identifiers so no information was available to identify the surgical patients from whom the tissue sections were derived. The patient studies were conducted in accordance with the following ethical guidelines: Declaration of Helsinki, International Ethical Guidelines for Biomedical Research Involving Human Subjects (CIOMS), Belmont Report, and US Common Rule.

**Figure 1.**
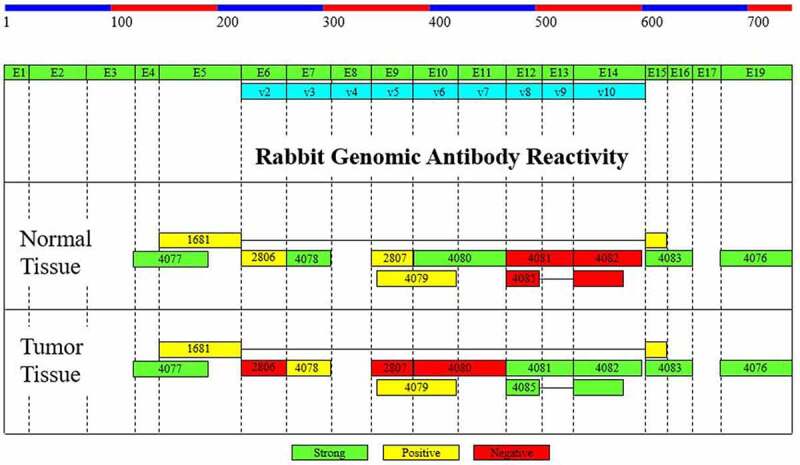
*Exon map of human CD44 isoforms showing regions of rabbit genomic antibody (RGA) binding and immunohistochemical (IHC) reactivity*. The anti-CD44 rabbit genomic antibodies were generated to react to 16 different epitopes that span the entire length of the CD44 molecule. Antibody 4077, which binds to CD44’s amino-portion, will react to all forms of CD44 including the standard CD44 isoform and all variant CD44 isoforms^35^ except CD44v2. Antibody 4080 will react to CD44v6+ cells and antibody 4081 will recognize CD44v8–10+ cells. Exon numbering is based on Screaton et al.^31^ and Uniprot^32^.

*DNA-immunization Genomic Technology Generated Anti-CD44Antibodies*. Rabbit anti-human CD44 genomic antibodies were generated at Strategic Diagnostics Inc. (SDIX, Newark, DE) using a proprietary technology developed at SDIX^30^. The anti-CD44 rabbit genomic antibodies were designed to react to different epitopes along the entire length of the CD44 molecule (Figure 1, Table S1). The reactivity of each antibody to human CD44 antigens was measured at SDIX Inc by sandwich ELISA immunoassay and high-throughput flow cytometry screening using NEK293 cells (Figures S1 S2). We used the established standard approach to systematically evaluate antibodies^30^. The approach employed several different assay technologies in a sequential fashion which is typically used to generate antibodies made by DNA immunization technology^30^. This stepwise screening process was designed in a way to identify which antibodies have high specificity and sensitivity by using IHC, flow cytometry, and sandwich immunoassays. Once candidate antibodies were selected using this screening approach, they were then validated using immunohistochemical and immunofluorescence staining on pathology samples of matched pairs of normal and malignant colonic tissues as described below.

*Immunohistochemical (IHC) and Immunofluorescence (IF) Analyses of Anti-CD44 Antibodies*. The rabbit anti-human CD44 genomic antibodies were analyzed to evaluate the expressionof different CD44 isoforms in malignant colonic tissue and adjacent normal mucosa using IHC and IF staining. Staining was done using paraffin-embedded sections of matched normal and tumor tissue of the colon (10 patients for each antibody). Figure 1 lists the regions of rabbit-generated genomic antibody (RGA) reactivity. In addition to our newly generated anti-CD44 RGA antibodies, we used anti-ALDH1 (BD Biosciences), anti-CD44v9 (BioLegend), and anti-LGR5 (Novus Biologicals) antibodies in our study. To validate that CD44v8–10 is expressed in SCs, an immunostaining experiment was done to show that CD44v8–10 is co-expressed with the ALDH1 SC marker in normal and malignant colon tissues. IHC and IF staining experiments were done as we previously described^2,33,34^. The immunostained specimens were imaged using a 20× objective of a ZEISS Epi-fluorescence microscope (20×) and analyzed using Zen software.

*Flow Cytometry*. CD44v8–10 mRNA expression was measured in LGR5+ cells compared to LGR5– cells. Results were obtained by sorting LGR5+ and LGR5– cells from the HT29 CRC cell line using fluorescence activated cell sorting (FACS). Sorted subpopulations were subjected to real-time quantitative PCR using primer pairs: forward 5’-GAC AGA ATC CCT GCTACC AATA-3’ and reverse 5’-ATG TGT CTT GGT CTC CTGATAA-3’ (DOI: 10.3892/ol.2016.4985). Cycling threshold (Ct) values were normalized to the GAPDH housekeeping gene. Fold change was determined using the formula: 2^average ∆Ct LGR5+ divided by 2^average ∆Ct LGR5–. Error bars were calculated using the standard error of the fold change mean of four replicates.

*Bioinformatics Analyses & Statistics*. Bioinformatics analysis on CD44’s ability to predict CRC patient survival was done through The Human Protein Atlas (https://www.proteinatlas.org). The data analyzed in the Pathology Atlas provides a correlation of mRNA expression and patient survival. Correlation analysis is based on CD44 expression levels in cancer tissue and clinical outcome (survival) for 597 CRC patients. The Kaplan-Meier plot summarizes results from the analysis of RNA-seq data generated by The Cancer Genome Atlas (TCGA). Statistics was based on t-test and Log-rank *p*-value.

## Results

**Figure 2.**
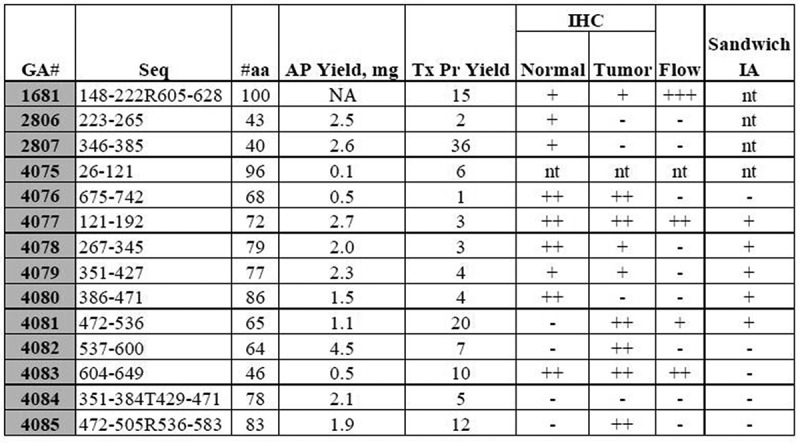
*Summary of screening results on the panel of anti-CD44 rabbit genomic antibodies*. Flow cytometric analyses showed that four antibodies (1681, 4077, 4081, 4083) had strong reactivity to their corresponding antigens (Figure S2). Sandwich ELISA immunoassays showed that five antibodies (4077–4081) had reactivity to antigens. IHC staining was used to screen reactivity against matched normal versus malignant colonic tissue samples. We found that: *i*) Three antibodies (4076, 4077, 4083) stained both normal colonic epithelial and colon carcinoma tissues; *ii*) Three antibodies (4081, 4082, 4085) strongly stained colon carcinomas, but did not stain normal colonic epithelium; *iii*). One antibody (4080) stained normal colonic epithelium, but not colon carcinoma. GA = genomic antibody, Seq = AA sequence, #aa = number of amino acids, AP = affinity purified, Tx Pr = direct bind ELISA, IHC = immunohistochemistry, Flow = flow cytometry, IA = immunoassay

*Generation and Screening of anti-CD44 Rabbit Genomic Antibodies*. A panel of anti-CD44 rabbit genomic antibodies was generated to 16 specific epitopes that span the entire length of the CD44 molecule (Figures 1 & 2, Table S1). Flow cytometric analyses showed that four antibodies (1681, 4077, 4081, 4083) had strong reactivity to their corresponding antigens (Figure S2). Sandwich immunoassays showed that five antibodies (4077–4081) had reactivity to antigens. We then performed, using IHC staining, an initial screening of the 16 antibodies to determine which of them stained normal versus malignant colonic SCs (Figure 2). We found that three antibodies (4076, 4077, 4083) stained normal colonic epithelium as well as colon carcinomas. In contrast, we found that three antibodies (4081, 4082, 4085) strongly stained colon carcinomas, but did not stain the normal colonic epithelium. One antibody (4080) stained a normal colonic epithelium, but not a colon carcinoma. Thus, these results show that our anti-CD44 rabbit genomic antibodies that are designed to react to different epitopes within the CD44 molecule appear to have different staining patterns in normal and malignant colon tissues.

*Ranking of anti-CD44 Antibodies for Further Study*. Based on the results from IHC, flow cytometry, and sandwich immunoassay analyses, three antibodies (4077, 4080, 4081) were selected for further analysis using immunofluorescence staining. Overall, results from our initial IHC screening showed that antibody 4077 stains both normal colon and CRC, antibody 4080 selectively stains normal colonic epithelium, and antibody 4081 preferentially stains CRC tissue. Based on the specific epitopes that the anti-CD44 rabbit genomic antibodies were designed to react to within the CD44 molecule, it can be determined which of these different antibodies recognize CD44’s different isoforms. Antibody 4077, which binds to CD44’s amino-portion, will recognize all forms of CD44 including the standard CD44 isoform (CD44s) and all variant CD44 isoforms (except CD44v2). Antibody 4080 will recognize the CD44v6 isoform, and antibody 4081 will recognize the CD44v8–10 isoform.

**Figure 3.**
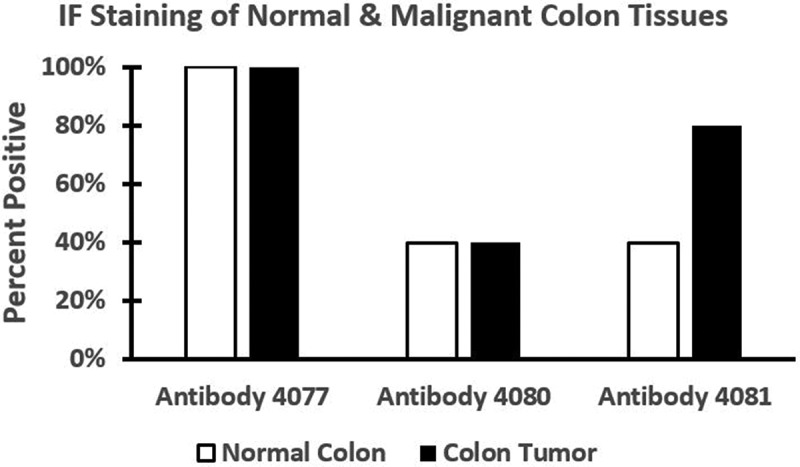
*The frequency of immunofluorescence staining with the different anti-CD44 antibodies*. Antibody 4077, designed to react to all forms of CD44 (except CD44v2), consistently (100%) stained both normal and malignant colonic tissues. Antibody 4080, designed to react to CD44v6, stained both normal and malignant colonic tissues in (40%) of cases. Antibody 4081, designed to react to CD44v8–10, frequently (80%) stained colon carcinomas, but normal colonic epithelium stained less frequently (40%). When our two sets of immunostaining data (IHC & IF, *n* = 10 matched normal/tumor tissue pairs) were taken into account, the staining difference between normal colon and CRC was significant (*p* = .037) for the antibody (4081) against CD44v8–10, but not for antibodies (4077 & 4080) against CD44v6 and pan-CD44.

**Figure 4.**
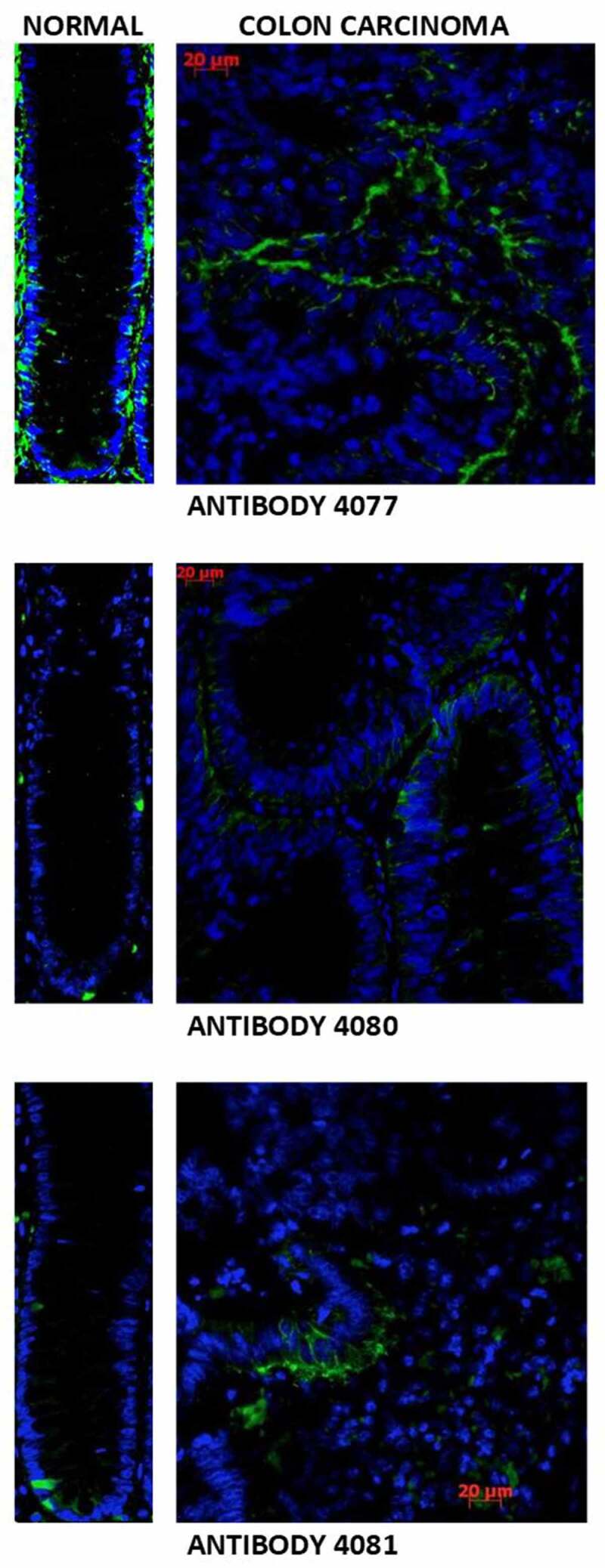
*The patterns of immunofluorescence staining using the different anti-CD44 antibodies*. Immunofluorescence staining of normal colonic epithelium using antibody 4077 (against all forms of CD44, except CD44v2) showed strong staining of the bottom 1/3 of the crypt as well as of the surrounding stroma. In CRC, antibody 4077 stained both colon carcinoma tissue and tumor stroma. Testing antibody 4080 (against CD44v6) on normal colonic epithelium showed staining of isolated cells in the lower crypt and some cells in the upper crypt; stroma staining was slight. For CRC, antibody 4080 stained both the colonic carcinoma and stromal tissues. Testing antibody 4081 (against CD44v8–10) on normal colonic epithelium showed staining of isolated cells in the crypt SC niche and stromal staining was negligible. For CRC, antibody 4081 typically stained colonic carcinoma tissue with only slight stromal tissue staining.

*Immunofluorescence Staining of Normal and Malignant Human Colon Tissues*. Five matched pairs of normal and malignant colonic tissues were then evaluated using these three different antibodies and immunofluorescence staining was done for CD44 expression. Figures 3 and 4 show the frequency and pattern of staining found with these different anti-CD44 antibodies. Our analysis of these three antibodies, which we designed to react to different CD44 epitopes, showed that they have different reactivity to normal versus malignant colonic tissues (described below).

*Antibody 4077 that Recognizes CD44s*. Antibody 4077 consistently (100%) stained both normal and malignant colonic tissues. Staining normal colonic epithelium with antibody 4077 showed that there was strong staining of the bottom 1/3 of the crypt as well as of the surrounding stroma. In CRC, antibody 4077 stained both colon carcinoma tissue and tumor stroma.

*Antibody 4080 that Recognizes CD44v6*. Antibody 4080 stained both normal and malignant colonic tissues in a minority (40%) of cases. In normal colonic epithelium, antibody 4080 stained isolated cells in the lower crypt and some cells in the upper crypt. With antibody 4080, stroma staining was slight. For CRC, antibody 4080 stained both the colonic carcinoma and stromal tissues.

*Antibody 4081 that Recognizes CD44v8–10*. Antibody 4081 frequently (80%) stained colon carcinoma and stained normal colonic epithelium less frequently (40%). For normal colonic epithelium, antibody 4081 stained isolated cells in the crypt SC niche and stromal staining was negligible. For CRC, antibody 4081 typically stained colonic carcinoma tissue with only slight stromal tissue staining.

**Figure 5.**
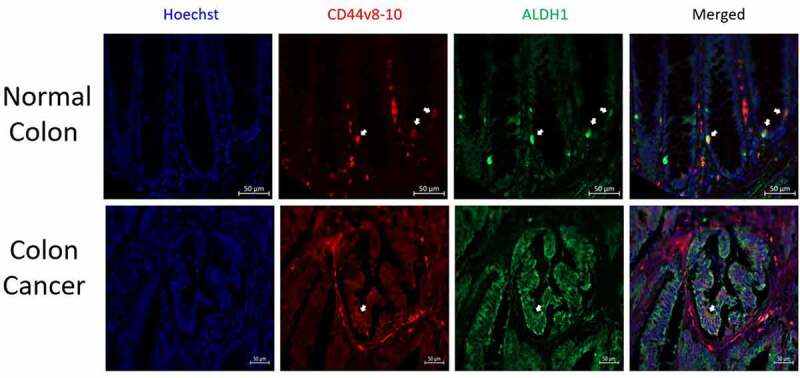
*Co-expression of CD44v8–10 and ALDH1 in normal human colon crypts and matched colon cancer tissue*. Immunofluorescent staining of normal colon and matched adenocarcinoma, (Stage 2A, descending colon) with anti-CD44v8–10 (red) and anti-ALDH1 (green) antibodies. Co-stained cells (yellow) are marked with white arrows. Imaging was done using a Zeiss Fluorescent Microscope (20×) and analyzed using Zen software.

*Validating that CD44v8–10 Marks Colonic SCs*. The expression of CD44v8–10 in SCs was validated using two different known SC markers, ALDH1, and LGR5. Immunostaining analysis showed that CD44v8–10 is co-expressed with the ALDH1 SC marker in normal and malignant colon tissues (Figure 5). Another widely studied SC marker is LGR5. However, immunostaining for LGR5 previously showed such low levels of LGR5 expression that results from immunostaining cannot be reliably interpreted^12^. Thus, we resorted to measuring LGR5 levels in LGR5+ cells isolated using FACS from a human CRC cell line and quantifying LGR5 expression by qPCR. We found that CD44v8–10 expression is increased 2.2-fold in LGR5+ cells compared to LGR5– cells (Figure S3).

**Figure 6.**
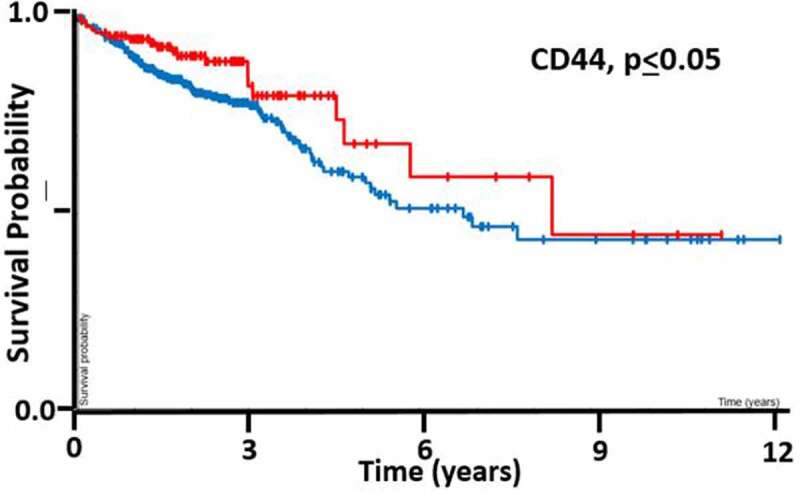
*Bioinformatics analysis of CD44's ability to predict CRC patient survival.* Bioinformatics results on analysis of CD44 expression levels and CRC patient survival (n = 597) was based on RNA-seq data generated by The Cancer Genome Atlas (TCGA) project. Note that, in the TCGA database, CD44 expression encompasses all forms of CD44 including standard CD44 and all CD44 variant isoforms [56] so it doesn't distinguish between CD44 isoforms. The Kaplan-Meier plot shows that CD44 expression correlates with CRC patient survival (p < 0.05).

*The Ability of CD44 to Predict CRC Patient Survival*. Bioinformatics analysis was also done on CD44’s ability to predict CRC patient survival using The Cancer Genome Atlas (TCGA) database. Unfortunately, the TCGA study on colon cancer using RNA-seq data doesn’t distinguish between the different CD44 isoforms so the correlation with CRC patient survival is for CD44s. Nonetheless, the Kaplan-Meier plot (Figure 6) does demonstrate that the overall expression of CD44 (including CD44 isoforms) correlates with the CRC patient survival (p<0.05).

## Discussion

Our goal was to investigate how different CD44 isoforms contribute to the emergence of CSC overpopulation that drives CRC development. Accordingly, we created a panel of anti-CD44 antibodies to specific epitopes within the CD44 molecule to study the expression of different CD44 isoforms in normal and malignant colonic tissues. Our IF staining of normal colonic epithelium indicates that both the variant CD44 isoforms CD44v6 and CD44v8–10 are expressed in normal colonic crypts. For the normal colonic epithelium, we found that both CD44v6+ and CD44v8–10+ cells are located in the crypt SC niche, but only a few CD44v6+ cells are also found in the upper crypt. This result suggests that CD44v8–10+ cells are predominantly colonic SCs. Our IF staining also showed that colon carcinoma tissues frequently (80%) stained for CD44v8–10, but less frequently (40%) for CD44v6. These results suggest that CD44v8–10 is the predominant CD44 variant isoform expressed in CSCs in colonic malignancies. They also show that CD44v8–10+ CSCs become overpopulated during colon cancer development. Thus, these findings on CD44v8–10 support our hypothesis that “specific CD44 variant isoforms are selectively expressed in normal colonic SCs and become overexpressed in CSCs during CRC development”.

In comparison, the antibody (4077) against the amino-portion of CD44 that recognizes all CD44 isoforms (except CD44v2) frequently (100%) stained both normal and malignant colonic tissues. However, in the normal colon, staining with this antibody extended beyond the SC niche into the proliferative crypt zone because CD44+ cells were found throughout the bottom and middle regions of the colonic crypt. We previously reported^2^ a similar staining pattern using an antibody against the standard form of CD44 (CD44s). That CD44s is not only expressed in normal and malignant colonic SCs but also, it is expressed in proliferative non-SCs indicates that CD44s is not a specific marker for colonic SCs. Consequently, we surmise that many other CD44 isoforms exist that are not specifically expressed in colonic SCs, i.e. these putative isoforms are expressed in non-SCs. Still, our bioinformatics analysis (Figure 6) based on the TCGA database that measures the level of all CD44 isoforms reveals that CD44 expression is correlated with CRC patient survival. However, a meta-analysis of CD44 expression in CRCs (*n* = 3,098 patients) revealed that CD44 is not a prognostic marker for CRC patient survival^36^. In comparison, Yamaguchi et al.^37^ reported that CD44v8–10 is an independent factor for predicting CRC patient prognosis. Thus, since we demonstrate that the variant CD44 isoform CD44v8–10 is selectively expressed in the colonic SCs, we predict that CD44v8–10 will be a more specific and better prognostic SC marker than pan-CD44 for CRC patient outcomes.

In previous studies, CD44v8–10 (also known as epithelial CD44 or CD44E) was discovered to be the major CD44 isoform expressed in epithelial cells^20,38,39^. However, much less is known about the role of CD44v8–10 in stem cells. Nonetheless, increased expression of CD44v8–10 does occur in many cancer types including gastric^40,41^, pancreatic^26^, hematologic (CML)^42^, breast^43^, bladder^44^, biliary tract^45,46^, thyroid^47^, esophageal^48^, ovarian^49^, and colorectal^37,50^ malignancies.

In gastric cancer, CD44v8–10 expression was shown to contribute to the initiation of gastric tumors^17,40,41^. In these studies, gain- and loss-of-function examination of CD44v8–10 showed that injection of CD44v8–10-depleted cells didn’t develop tumors compared to CD44v8–10-positive cells. Moreover, CD44v8–10, but not CD44s, rescued the tumor-initiating potential of the CD44-depleted gastric cells. These findings indicate that CD44v8–10 is a cancer-specific marker for gastric CSCs^17,40,41^.

The role of CD44v8–10 in colon cancer development has been understudied. One recent study by Dastych et al.^51^ showed that CD44v8–10 is frequently (92%) overexpressed in early colonic adenomas. Given the following: *i*) we found that CD44v8–10+ cells are located in the normal human colonic SC niche; *ii*) CD44v8–10 is overexpressed in premalignant colonic tumors^51;^ *iii*) we found that CD44v8–10+ CSCs become overpopulated during CRC progression, we conclude that CD44v8–10 likely contributes to the SC overpopulation that drives the development and growth of colon cancers. Overall, the expression of CD44v8–10 has vast clinical significance because many cancer types express this variant isoform, and because CD44v8–10 promotes resistance to cancer therapy^52–56^. Since the CD44 variant v8–10 epitope is located on CD44’s extracellular region, it offers great promise for targeted anti-CSC treatment approaches.

## Supplementary Material

Supplemental Material
