# Supplementary material for: The v8-10 variant isoform of CD44 is selectively expressed in the normal human colonic stem cell niche and frequently is overexpressed in colon carcinomas during tumor development
Source: Cancer Biol Ther. 2023 Apr 2;24(1):2195363. doi: 10.1080/15384047.2023.2195363 (PMC10072056; doi:10.1080/15384047.2023.2195363)
Supplement: Supplemental Material [file KCBT_A_2195363_SM9149.pdf]

Supplemental Material

Table S1. CD44 Exons. Numbering from Screaton et al. PNAS 89:12160-4, 1992.

Exon Screaton *et al.*	Exon pre-mRNA	#aa	Aa seq position in P16070	Variable exon	Aa Sequence from Screaton *et al.* PNAS, 1992
1	1	23	1-23		MDKFWWHAAWGLCLVPLSLAQID
2	2	55	24-78		LNITCRFAGVFHVEKNGRYSISRTEAADLCKAFNSTLPTMAQM EKALSIGFETCR
3	3	45	79-123		YGFIEGHVVIPRIHPNSICAANNTGVYILTSNTSQYDTYCFNASA
4	4	23	124-146		PPEEDCTSVTDLPNAFDGPITIT
5	5	77	147-223		IVNRDGTRYVQKGEYRTNPEDIYPSNPTDDDVSSGSSSERSSTSG GYIFYTFSTVHPIPDEDSPWITDSTDRIPATT
-	6			V1	
6	7	43	224-266	V2	LMSTSATATETATKRQETWDWFSWLFLPSESKNHLHTTTQMA G
7	8	42	267-308	V3	TSSNTISAGWEPNEENEDERDRHLSFSGSGIDDDEDFISSTI
8	9	38	309-346	V4	STTPRAFDHTKQNQDWTQWNPSHSNPEVLLQTTTRMTD
9	10	39	347-385	V5	VDRNGTTAYEGNWNPEAHPPLIHHEHHEEEETPHSTSTI
10	11	43	386-428	V6	QATPSSTTEETATQKEQWFGNRWHEGYRQTPKEDSHSTTGTAA
11	12	44	429-472	V7	ASAHTSHPMQGRTTPSPEDSSWTDFFNPISHPMGRGHQAGRRM D
12	13	34	473-506	V8	MDSSHSITLQPTANPNTGLVEDLDRTGPLSMTTQ
13	14	30	507-536	V9	QSNSQSFSTSHEGLEEDKDHPTTSTLTSSN
14	15	68	537-604	V10	RNDVTGGRRDPNHSEGSTTLLEGYTSHYPHTKESRTFIPVTSAK TGSFGVTAVTVGDSNSNVNRSLSG
15	16	21	605-625		DQDTFHPSGGSHTTHGSESDG
16	17	24	626-649		HSHGSQEGGANTTSGPIRTPQIPE
17	18	26	650-675		WLIILASLLALALILAVCIAVNSRRR
18	19				
19	20	67	676-742		CGQKKKLVINSGNGAVEDRKPSGLNGEASKSQEMVHLVNKES SETPDQFMTADETRNLQNVDMKIGV

CD44 RGA Titers

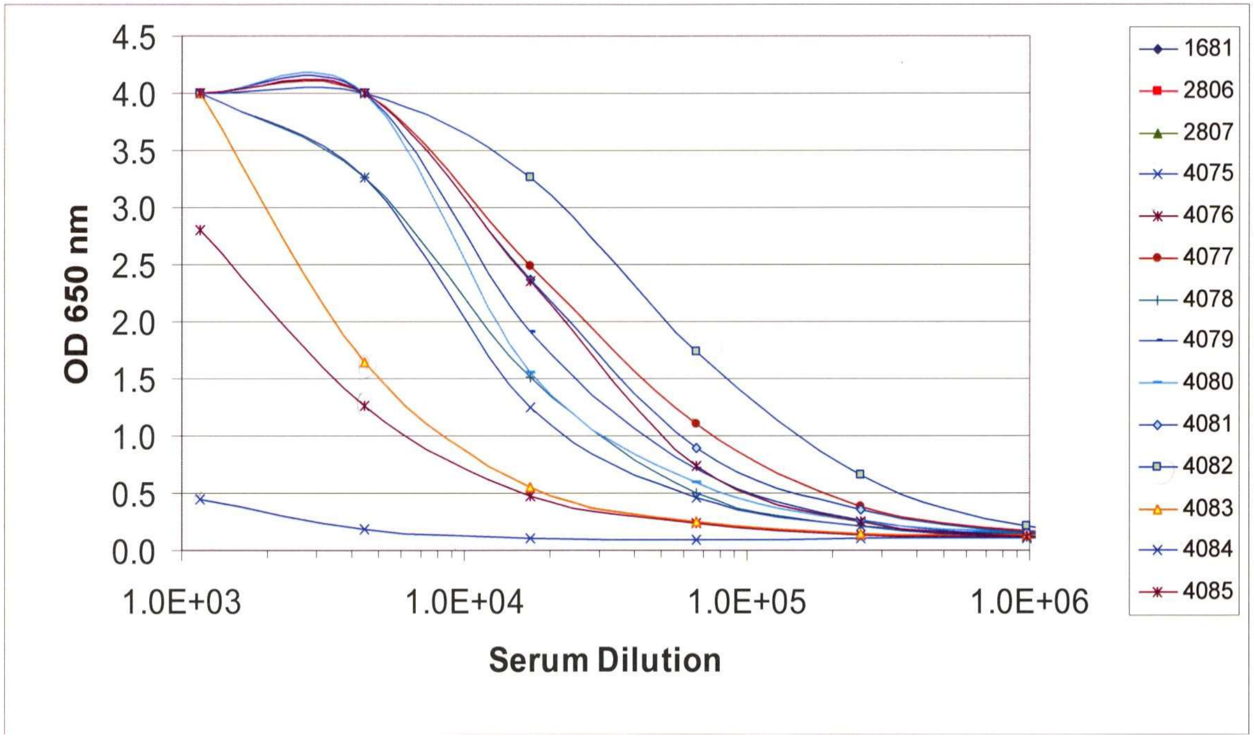

Figure S1. **CD44 rabbit genomic antibody (RGA) titers**. All newly generated antibodies were titered against the protein fragment encoded by the respective DNA sequence.

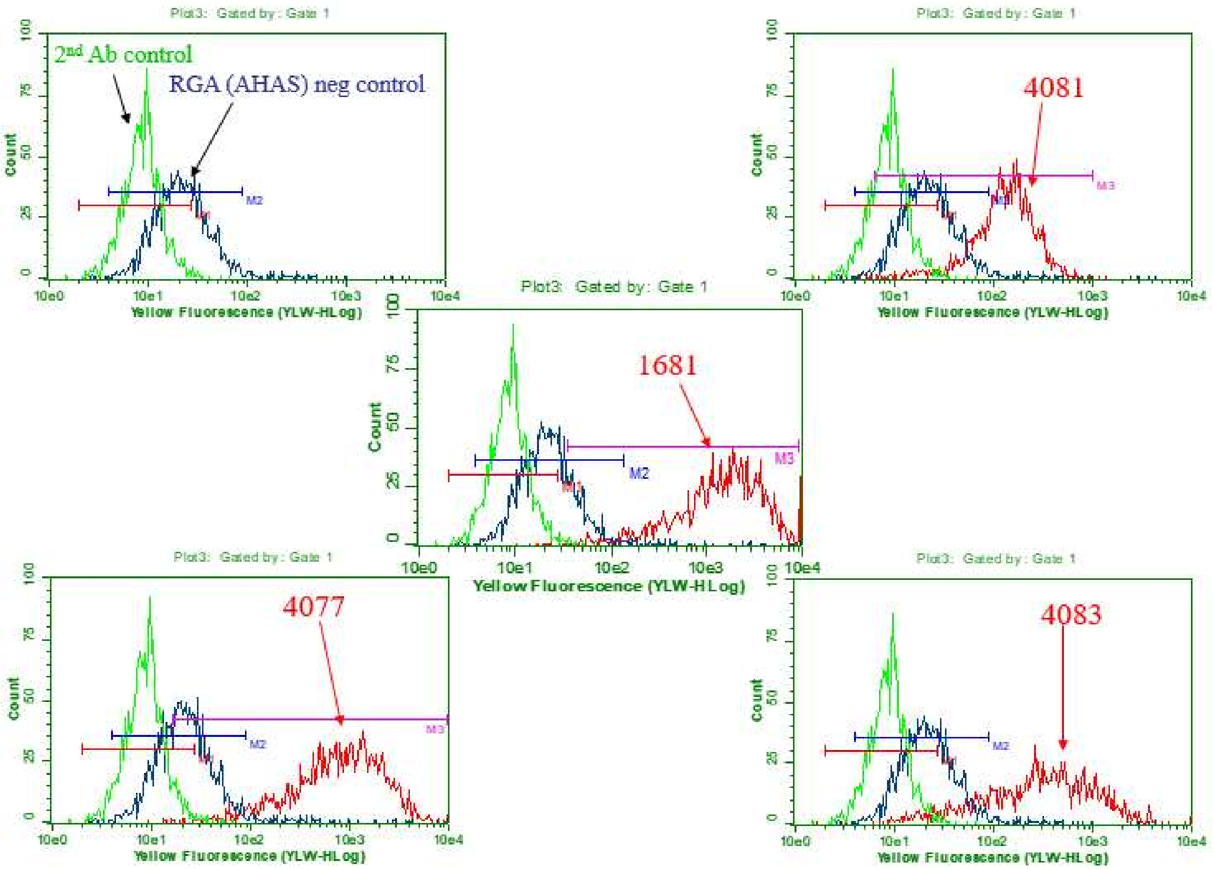

Figure S2. **Analysis of anti-CD44 rabbit genomic antibodies by flow cytometry**. The reactivity of each antibody to human CD44 antigens was measured high-throughput flow cytometry screening using NEK293 cells. Flow cytometric analyses showed that four antibodies (1681, 4077, 4081, 4083) had strong reactivity to their corresponding antigens. The control is shown in upper left panel.

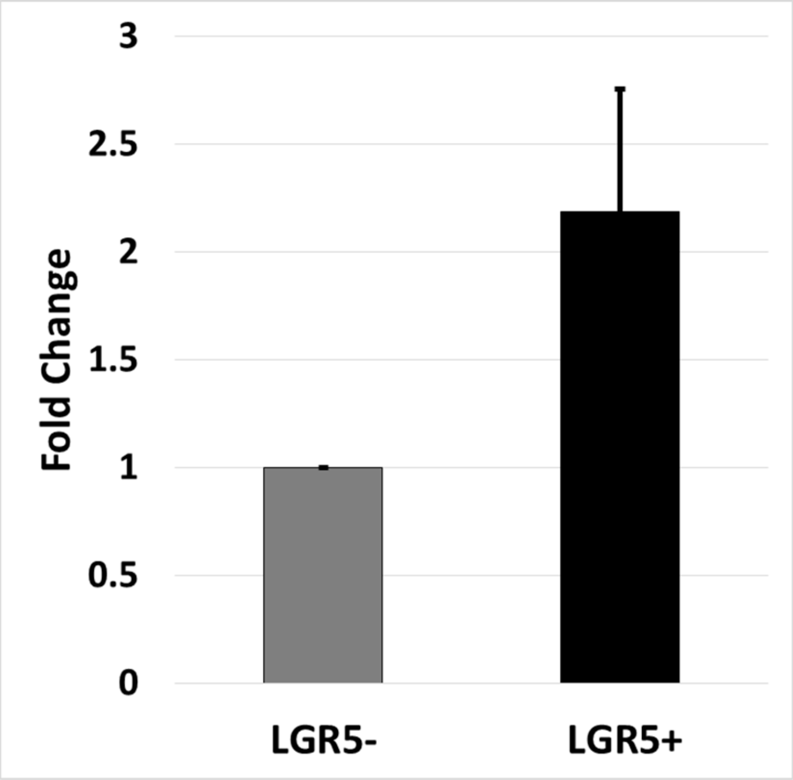

Figure S3. **CD44v8-10 mRNA expression in LGR5+ versus LGR5 –cells**. LGR5+ and LGR5 –cells were isolated from human HT29 CRC cells using (FACS) and analyzed by qPCR. CD44v8-10 expression is 2.2 fold increased in LGR5+ cells compared to LGR5 –cells. Error bars were calculated using standard error of the fold change mean of 4 replicates.
